# Additional risk stratification in women with a history of spontaneous preterm birth and a midtrimester cervical length > 25 millimeters

**DOI:** 10.1007/s00404-026-08364-9

**Published:** 2026-02-23

**Authors:** Emilie V. J. van Limburg Stirum, Sofie H. Breuking, Charlotte E. van Dijk, Janneke van ’t Hooft, Brenda M. Kazemier, Annemijn A. de Ruigh, Martijn A. Oudijk, Eva Pajkrt, Marjon A. de Boer

**Affiliations:** 1 Department of Obstetrics and Gynaecology, Amsterdam UMC, University of Amsterdam, Meibergdreef 9, 1105 AZ Amsterdam, The Netherlands; 2 Amsterdam Reproduction and Development Research Institute, Amsterdam, The Netherlands; 3 Department of Obstetrics and Gynaecology, Amsterdam UMC, Vrije Universiteit Amsterdam, de Boelelaan 1117, Amsterdam, The Netherlands

**Keywords:** Cervical length, Cutoff value, Cervical shortening, Recurrent spontaneous preterm birth

## Abstract

**Purpose:**

To determine whether there is an association between cervical length > 25 millimeters (mm) and a decrease in cervical length before 24 weeks of gestation with an increased risk of recurrent spontaneous preterm birth (sPTB).”

**Methods:**

This retrospective cohort study includes women with a singleton pregnancy, a previous sPTB before 34 weeks of gestation, serial cervical length measurements and a shortest midpregnancy cervical length of > 25 mm. Participants received care according to local protocols for the prevention of preterm birth in two academic hospitals in the Netherlands between February 2005 and September 2021. Exclusion criteria were fetal structural anomalies, signs of threatened preterm birth or treatment with a cerclage or pessary. Cervical length measurements were grouped in three timepoints in pregnancy that were chosen a priori: 14 + 0 to 18 + 6 weeks (CL1); 19 + 0 to 20 + 6 weeks (CL2); and 21 + 0 to 23 + 6 weeks (CL3). Outcome measures included percentage of sPTB (< 37, < 34 and < 28 weeks of gestation) with 95% confidence intervals (95% CI) and Odds Ratio’s (OR). Association between decrease in cervical length and sPTB were calculated using logistic regression.

**Results:**

In total, 469 pregnancies were included. Overall, sPTB recurred in 21.1% (95% CI 17.4–24.8%), 9.0% (95% CI 6.4–11.6%) and 1.9% (95% CI 0.7–3.2%) before 37, 34 and 28 weeks of gestation, respectively. Women with a cervical length of > 25–30 mm in CL3 were at higher risk to deliver before 37 weeks, compared to women with a cervical length > 30 mm (44.7% versus 18.5%, OR 3.6, 95% CI 1.91–6.66). The decrease in cervical length between timepoint CL1, CL2 and CL3 had no association with a recurrent sPTB.

**Conclusions:**

Women with a history of sPTB before 34 weeks of GA and a cervical length of > 25–30 mm before the 24 weeks of gestation have an almost four times higher risk for a recurrent sPTB, compared to those with a longer cervical length. No association was found between decrease in cervical length and the risk of sPTB. Future studies should assess whether women with a history of sPTB and a cervical length of > 25–30 mm benefit from cerclage and cutoff values could be revised accordingly.

**Supplementary Information:**

The online version contains supplementary material available at 10.1007/s00404-026-08364-9.

## What does this study add to the clinical work

**Table:** 

Women with a history of spontaneous preterm birth (sPTB) and a shortest midtrimester cervical length >30 mm can be reassured that their risk to deliver preterm does not further increase from baseline risk after a previous sPTB. Women with a previous sPTB and a midpregnancy cervical length of >25-30 mm are at high risk of recurrent sPTB (44.7%), and future studies should investigate whether interventions, such as cerclage, are beneficial in this group.	

## Introduction

Pregnant women with a history of a spontaneous preterm birth (sPTB) have a five times elevated risk for a recurrent sPTB in a subsequent pregnancy [1]. In women with a short cervix (i.e., 25 millimeters [mm] or less), the risk for a recurrent sPTB may increase even further [2]. Therefore, management of patients with a history of sPTB includes serial cervical length measurements starting from 14 or 16 weeks up to 24 weeks of gestation [3]. In case of a cervical length ≤ 25 mm, a cerclage may be effective in reducing sPTB [3, 4].

In case of a cervix > 25 mm, no cerclage is offered. However, women with a history of sPTB and a cervical length > 25 mm remain at risk for a recurrent sPTB. Although this group of women receive repeated cervical length measurement between 14–16 and 24 weeks of gestation as standard care, the information of cervical length change is not used for stratification of these woman with a substantially increased risk. We hypothesize that there might be a subgroup of women with a cervix > 25 mm in the second trimester that might also benefit from cerclage treatment.

Several studies have explored the predictive value of cervical length decrease, but evidence is inconsistent. The systematic review and meta-analysis of Conde-Agudelo et al. (2015) concluded that any cervical change over time has a lower predictive capacity for sPTB than a single measurement between 18 and 24 weeks. [5] However, decrease in millimeters of the cervix was not specified. Literature reporting on cervical shortening in women with a history of sPTB is scarce. This leaves a gap in understanding the precise impact of cervical length decrease in this population and in possible inaccurate identification of women at increased risk. Therefore, this study aims to determine if there is an association between a decrease in cervical length and a recurrent sPTB in women with a prior sPTB and a cervical length > 25 mm before 24 weeks of gestation.

## Methods

This is a retrospective cohort study using data from two academic hospitals in the Netherlands from February 2005 to September 2021. The Medical Ethics Committee of the Amsterdam UMC deemed that the Medical Research Involving Human Subjects Act did not apply to this study (W21_218#21.237). The manuscript is reported following Strengthening the Reporting of Observational Studies in Epidemiology (STROBE) guidelines.

### Data collection

We conducted a search in the electronic ultrasound database *Astraia software* to identify women eligible for this study at both locations. Women with a history of sPTB before 34 weeks of gestation (i.e., that started with spontaneous contractions or spontaneous rupture of the membranes), who in a subsequent singleton pregnancy had multiple cervical length measurements before 24 weeks of gestation, all > 25 mm, were eligible for inclusion. All women were offered standardized management according to the local protocol and gave consent to use their data for research purposes. Serial transvaginal cervical length measurements were performed starting from 14 weeks until 24 weeks of gestation and based on their obstetric history all women were counseled for preventive treatment with progestagens (i.e., Utrogestan 200 mg vaginally daily or weekly intramuscular injections with 250 mg 17-α-hydroxyprogesterone caproate from 16 until 36 weeks of gestation). If a cervix below 25 mm was detected before 24 weeks of gestation, placement of a cerclage or randomization in a randomized controlled trial (assessing a pessary versus a cerclage) was offered [6]. All cervical measurements were performed by experienced and trained sonographers following the Fetal Maternal Foundation guidelines. [7]

Exclusion criteria were age below 18 years, a cervical length ≤ 25 mm in any timepoint, multiple pregnancy, fetal structural anomalies visible at ultrasound, signs of threatened preterm birth before 24 weeks of gestations (e.g., contractions or vaginal bleeding), placement of a cerclage or cervical pessary, or missing data regarding cervical length measurements or pregnancy outcomes.

By chart review, we collected baseline characteristics, obstetric and medical history, pregnancy interventions, and obstetrical and neonatal outcomes. For each woman, cervical length measurements were collected at three different timepoints in pregnancy [between 14 + 0–18 + 6 weeks (CL1), 19 + 0–20 + 6 weeks (CL2), and 21 + 0–23 + 6 weeks (CL3)]. For women who had more than one pregnancy during the study period, the pregnancy with measurements taken at all three timepoints (CL1–3) was included (and in case of multiple available pregnancies, the most recent one). In cases of multiple measurements within the same timeframe, the shortest cervical length measurement was used. Cervical length shortening was documented per millimeter and defined as at least 1 mm shorter as compared to a previous measurement. We reported cervical length shortening using clinical cutoff points of 5 and 10 mm or more.

### Outcome measures

The primary outcome was sPTB, defined as birth < 37 weeks of gestation that started with spontaneous contractions or spontaneous rupture of the membranes. Thereby, the association between decrease in cervical length in the second trimester and sPTB before 37, 34 and 28 weeks was assessed.

### Statistical analyses

Baseline characteristics were presented as continuous or dichotomous outcome as appropriate. The risk for sPTB for different cutoff points of cervical length (i.e., > 25–30 mm, > 30–35 mm and > 35 mm) at different timepoints in pregnancy (CL1–3) was shown in percentages. Association between the decrease in cervical length and sPTB before 37, 34 and 28 weeks of gestation were assessed by a logistic regression. Outcomes were presented as Odds Ratio (OR) with corresponding 95% CI. The risk for sPTB was compared between cervical length shortening between CL1 and CL3 weeks and the absolute cervical length in CL3. A subgroup analysis assessing the association between the decrease in cervical length and sPTB was conducted for women who had received progesterone treatment. Statistical analyses were performed using Statistical Package for Social Science version 28; IBM, Endicott, NY.

## Results

**Fig. 1:**
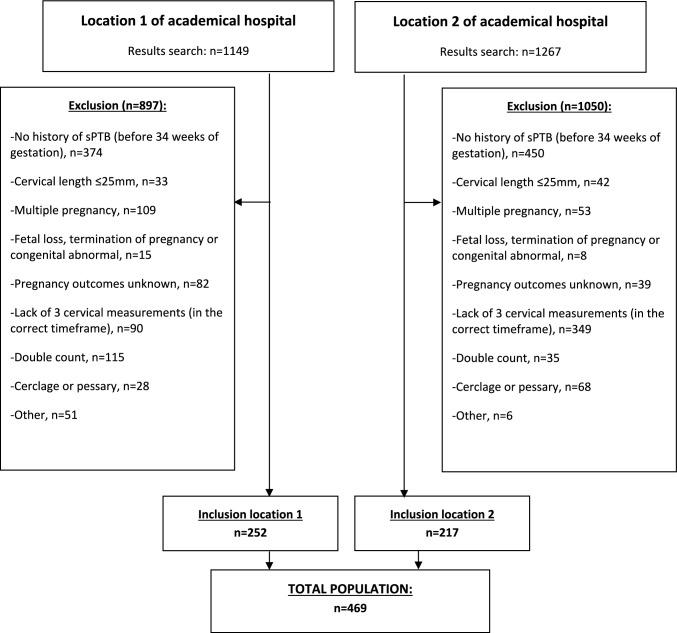
Flowchart. * If there was more than one pregnancy during the study period, the most recent pregnancy was included in this study

**Fig. 2:**
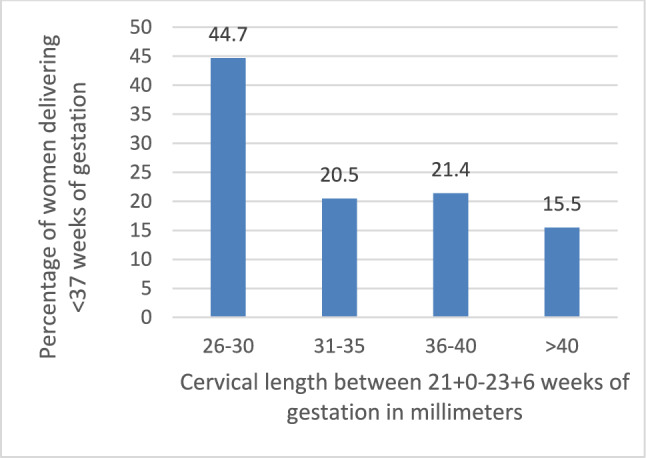
Percentage of women delivering before 37 weeks of gestation and their cervical length between 21 + 0 and 23 + 6 weeks of gestation

A total of 2416 women with a previous sPTB and who received cervical length measurements were identified of which 469 could be included (Fig. 1). Baseline characteristics are detailed in Table 1. The majority of women received progesterone (340/469, 72.5%), with an increase in use over the years. The mean cervical length was 40.6 mm (7.0 SD), 41.5 mm (7.4 SD) and 39.6 mm (7.1 SD) at CL1, CL2 and CL3, respectively. The distribution of the cervical length in CL3 can be found in Appendix S1. Overall, the risk of birth before 37, 34 and 28 weeks of gestation was 21.1% (95% CI 17.4–24.8), 9.0% (95% CI 6.4–11.6) and 1.9% (95% CI 0.7–3.2), respectively. Compared to women with a cervical length > 30 mm, women with a cervical length of > 25–30 mm at timepoint CL3 were at higher risk to deliver before 37 weeks of gestation (44.7% versus 18.5%, OR 3.6; 95% CI 1.91–6.66) (Tables 2, 3 and Fig. 2).

**Table 1:** Baseline characteristics

Baseline characteristics	Number of analyzed women	Total cohort with CL > 25 mm *n *= 469
Maternal age at delivery, mean ± SD	346	32.7 ± 4.6
Ethnic origin European, number (%)	412	265 (64.3)
BMI in kg/m2, median (IQR)	437	23.7 (21.5–26.7)
*Diabetes, number (%)*	457	
Gestational diabetes		28 (6.1)
Pre-existing		6 (1.3)
*Hypertension, number (%)*	462	
Pregnancy induced or pre-eclampsia		22 (4.7)
Pre-existing		5 (1.1)
*Smoking status, number (%)*	438	
Current smoker		16 (3.7)
Former smoker		32 (7.3)
*Obstetric history*	469	
1 sPTB		278 (59.3)
> 1 sPTB		56 (11.9)
1 sPTB and 1 or more term births		135 (28.8)
Curettage in history, number (%)	409	42 (10.3)
Uterine anomaly, number (%)	468	20 (4.3)
*Cervical surgery in history, number (%)*	468	
LETZ		18 (3.8)
Conisation		1 (0.2)
*Type of progesterone, number (%)*	469	
Intramuscular (Proluton®)		285 (60.8)
Vaginal tablets (Utrogestan®)		55 (11.7)
No progesterone use		129 (27.5)
*Received progesterone, number (%)*		
2005 – 2009	93	38 (40.9)
2010 – 2015	169	119 (70.4)
2016 – 2021	207	183 (88.4)
Total	469	340 (72.5)

*CL* cervical length, *BMI* body mass index, *SD* standard deviation, *IQR* interquartile range, *LETZ* loop excision of the transformation zone, *sPTB* spontaneous preterm birth

**Table 2:** Recurrent spontaneous preterm birth (sPTB) risk in women with a shortest cervical length > 25 mm before 24 weeks of gestation for different cutoffs of the cervical length measured at different timepoints in pregnancy

GA at measurement and used cutoff	sPTB < 37 weeks of gestation *n* = 99/469	sPTB < 34 weeks of gestation *n* = 42/469	sPTB < 28 weeks of gestation *n* = 9/469
*CL1*			
> 25–30 mm	43.3% (13/30)	20.0% (6/30)	6.7% (2/30)
> 30–35 mm	15.6% (12/77)	6.5% (5/77)	0% (0/77)
> 35 mm	20.4% (74/362)	8.6% (31/362)	1.9% (7/362)
*CL2*			
> 25–30 mm	28.6% (8/28)	10.7% (3/28)	3.6% (1/28)
> 30–35 mm	30.9% (21/68)	13.2% (9/68)	4.4% (3/68)
> 35 mm	18.8% (70/373)	8.0% (30/373)	1.3% (5/373)
*CL3*			
> 25–30 mm	44.7% (21/47)	23.4% (11/47)	6.4% (3/47)
> 30–35 mm	20.5% (17/83)	8.4% (7/83)	3.6% (3/83)
> 35 mm	18.0% (61/339)	7.1% (24/339)	0.9% (3/339)

*GA* gestational age, *sPTB* spontaneous preterm birth, *CL1* cervix measured between 14 + 0 and 18 + 6 weeks of gestation, *CL2* cervix measured between 19 + 0 and 20 + 6 weeks of gestation, *CL3* cervix measured between 21 + 0 and 23 + 6 weeks of gestation

**Table 3:** Cervical length versus cervical shortening and spontaneous preterm birth (sPTB) risk before 37 weeks of gestation in women with a history of sPTB and a shortest cervical length > 25 mm

sPTB before 37 weeks of gestation		Cervical shortening CL1 – CL3		
		≤ 10 mm *n* = 431	> 10 mm *n* = 38	Total
Single CL measurement in CL3	> 25–30 mm *n* = 47	13/30 (43.3%)	8/17 (47.1%)	21/47 (44.7%)
	> 30 mm *n* = 422	76/401 (19.0%)	2/21 (9.5%)	78/422 (18.5%)
	Total	89/431 (20.6%)	10/38 (26.3%)	99/469 (21.1%)

*sPTB* spontaneous preterm birth, *GA* gestational age, *CL* cervical length, *CL1* cervix measured between 14 + 0 and 18 + 6 weeks of gestation, *CL3* cervix measured between 21 + 0 and 23 + 6 weeks of gestation

The mean of cervical change between CL1 and CL2 was + 0.9 mm (7.5 SD), between CL1 and CL3 -0.9 mm (7.3 SD) and between CL2 and CL3 -1.8 mm (7.1 SD). Change in cervical length was not always consistent across the three timepoints (e.g., the cervix was sometimes measured shorter in CL2 compared to the measurement at timepoint CL3). There was no significant association found between change in cervical length across the three timepoints and the risk of preterm delivery before 37, 34, and 28 weeks of gestation (Table S1). The absolute cervical length was more associated with a sPTB than a decrease in cervical length (Table 3).

### Subgroup analyses

Preterm birth rate before 37 weeks of gestation with and without progesterone were 23.5% and 14.7%, respectively (Table S2). When assessing women who used progesterone compared to those who did not use progesterone, no association was found between sPTB and decrease in cervical length between CL1 and CL3 (Table S2).

## Discussion

In this study of 469 asymptomatic women with a history of sPTB, a singleton pregnancy and a shortest cervical length before 24 weeks of gestation > 25 mm, the risk for a recurrent sPTB was 21.1%. A small group of women, 10.0% of women included in this cohort, had a cervical length > 25–30 mm between 21 + 0 and 23 + 6 and had a very high risk of recurrent sPTB (44.7%). No association was found between cervical decrease and the risk for a sPTB in our assessed population.

Although the populations assessed are not directly comparable between studies, findings of this study are in line with the systematic review of Conde-Agudelo et al. (2015) that showed a low predictive capacity of the decrease in cervical length for PTB before 37 and 35 weeks of gestation in singletons [5]. Similar to their study, this study suggests that the absolute cervical length predicts sPTB better than a decrease in cervical length.

Previous literature report an absolute recurrence risk of 16% for women with a history of PTB and a cervical length ≥ 25 mm. [8] Similar, we reported a risk of 19% to deliver preterm for women with a cervix > 30 mm before 23 + 6 weeks of gestation. Decrease in cervical length did not affect this risk. Therefore, women with cervical shortening and a persistent cervix > 30 mm, can be reassured. The high risk we observed for women with a cervical length between > 25 and 30 mm (44.7%) might suggest that we incorrectly classify these women as being at lower risk for preterm birth compared to those with a cervical length ≤ 25 mm. The association between shorter cervical length and an increased risk of spontaneous preterm birth (PTB) follows a continuous scale. Our research suggests that the current cutoff value of 25 mm may need to be reconsidered. Future studies should assess whether women with a history of sPTB and a cervical length of > 25–30 mm benefit from a cerclage.

The mean cervical length in this study was 39.6 mm (7.1 SD) in CL3, which is longer than reported in previous literature (mean 30.1 ± 10.1 SD, measured between 21 and 24 weeks of gestation) [9]. This difference can be explained by the inclusion of only women with a cervical length > 25 mm or differences in ethnicities across studies.

We observed that the decrease in cervical length was the highest between timepoint CL2 to CL3. This emphasizes the importance of continuing cervical length measurements up to 21 + 0 and 23 + 6 weeks of gestation and treat accordingly if cervical length ≤ 25 mm. [4, 10] In literature it is debated if women could benefit from follow-up after 24 weeks of gestation. Although not significant, a recent systematic review showed a possible reduction in PTB with a cerclage in women with a history of PTB and a short cervix detected between 24 and 26 weeks of gestation. This suggests a potential benefit of extending the follow-up period and not limit cerclage treatment to 24 weeks of gestation. [11]

Strengths of this study include the assessment of a high-risk population where accurately identifying those at risk has the greatest potential to reduce recurrent sPTB. Our research adds to the scarce existing evidence focusing on the decrease in cervical length in women with a history of PTB and a persistent cervical length > 25 mm.

This study also has several limitations. First, potential bias and confounding can be present due to its retrospective design. Due to the reliance on existing data we were not able to verify data quality and consistency. Exclusion of women with no repeated cervical measurements may have introduced selection bias. Because cervical length measurements are not blinded, the cutoff of 25 mm could have influenced the measurement. During this study studies on obstetric interventions (e.g., cerclage or pessary) took place [6]. This could have unintentionally led to shorter measurements of the cervix to avoid withholding a patient from treatment, but also to overmeasurement of the cervix, leading to more women at risk for preterm birth in our > 25–30 mm subgroup and causing overestimation of our results [12]. However, since all cervical measurements were performed by experienced and trained sonographers, we expect that this has minimally influenced our results. Second, our population is limited to women with a persistent cervical length > 25 mm. Therefore, firm conclusions about the association between decrease in cervical length and preterm birth in general could not be drawn. Third, the origin of the data obtained varied from 2005 until 2021. It is possible that in the past, documentation in medical records was less accurate, resulting in under documentation of patients’ medical history or interventions received. Fourth, the change in cervical length was not always consistent across the three timepoints (e.g., sometimes the cervix was measured shorter in timepoint CL2 compared to timepoint CL3). This may be explained by interobserver variation or contractions of the lower uterine segment [13–16]

## Conclusion

Women with a history of sPTB and a shortest cervix of > 25–30 mm before 24 weeks of gestation, have a very high risk for a recurrent sPTB. No association between cervical decrease with a persistent cervical length > 25 mm and the risk for sPTB was found. The absolute cervical length is more associated with a sPTB than a decrease in cervical length. This study supports the current guidelines which advise to continue serial cervical length measurements at least up to 23 + 6 weeks of gestation. Future studies should assess whether women with a history of sPTB and a cervical length of > 25–30 mm benefit from an intervention, such as cerclage, and cutoff values could be revised accordingly.

## Supplementary Information

Below is the link to the electronic supplementary material.

Supplementary file1 (DOCX 31 KB)

## Data Availability

No datasets were generated or analysed during the current study.
