# Supplementary material for: Additional risk stratification in women with a history of spontaneous preterm birth and a midtrimester cervical length > 25 millimeters
Source: Arch Gynecol Obstet. 2026 Feb 23;313(1):106. doi: 10.1007/s00404-026-08364-9 (PMC12929326; doi:10.1007/s00404-026-08364-9)
Supplement: Supplementary file 1 — Supplementary file1 (DOCX 31 KB) [file 404_2026_8364_MOESM1_ESM.docx]

**Appendix S1.** Distribution of cervical length between 21+0-23+6 weeks of gestation in women with a history of sPTB and a shortest cervical length >25mm.
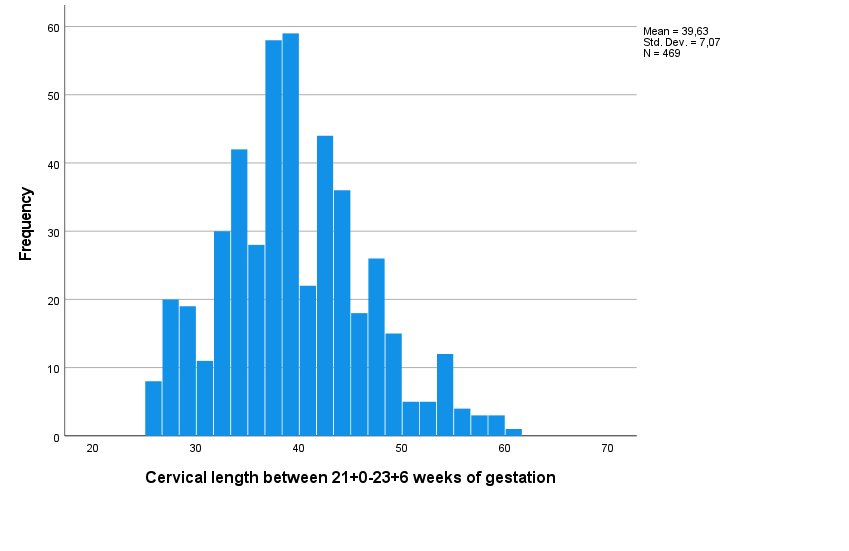


**Table S1.** Cervical shortening in different timeframes and the preterm birth risk in women with a history of spontaneous preterm birth and a shortest cervical length >25mm.

| **Total**  **n=469** | **sPTB<37 weeks of gestation**  **n=99** | **Odds Ratio (95%CI)** | **sPTB <34 weeks of gestation**  **n=42** | **Odds Ratio (95%CI)** | **sPTB <28 weeks of gestation n=9** | **Odds Ratio (95%CI)** |
| --- | --- | --- | --- | --- | --- | --- |
| **CL1-CL2** |  |  |  |  |  |  |
| No shortening, n (%)  n=247 | 49/247 (19.8) | 1.00 (ref.) | 20/247 (8.1) | 1.00 (ref.) | 4/247 (1.6) | 1.00 (ref.) |
| 1-10mm, n (%)  n=195 | 42/195 (21.5) | 1.11 (0.70-1.76) | 20/195 (10.3) | 1.30 (0.68-2.49) | 5/195 (2.6) | 1.60 (0.42-6.04) |
| >10 mm, n (%)  n=27 | 8/27 (29.6) | 1.70 (0.70-4.12) | 2/27 (7.4) | 0.91 (0.20-4.12) | 0 | n/a |
| **CL1-CL3** |  |  |  |  |  |  |
| No shortening, n (%) n=193 | 35/193 (18.1) | 1.00 (ref.) | 14/193 (7.3) | 1.00 (ref.) | 4/193 (2.1) | 1.00 (ref.) |
| 1-10mm, n (%)  n=238 | 54/238 (22.7) | 1.33 (0.82-2.13) | 23/238 (9.7) | 1.37 (0.68-2.74) | 3/238 (1.3) | 0.60 (0.13-2.73) |
| >10 mm, n (%)  n=38 | 10/38 (26.3) | 1.61 (0.72-3.62) | 5/38 (13.2) | 1.94 (0.65-5.74) | 2/38 (5.3) | 2.63 (0.46-14.87) |
| **CL2-CL3** |  |  |  |  |  |  |
| No shortening, n (%) n=163 | 27/163 (16.6) | 1.00 (ref.) | 14/163 (8.6) | 1.00 (ref.) | 4/163 (2.5) | 1.00 (ref.) |
| 1-10mm, n (%)  n=259 | 62/259 (23.9) | 1.59 (0.96-2.62) | 23/259 (8.9) | 1.04 (0.52-2.08) | 3/259 (1.2) | 0.47 (0.10-2.11) |
| >10 mm, n (%)  n=47 | 10/47 (21.3) | 1.36 (0.61-3.07) | 5/47 (10.6) | 1.27 (0.43-3.72) | 2/47 (4.3) | 1.77 (0.31-9.96) |

sPTB: spontaneous preterm birth. CL1: cervix measured between 14+0-18+6 weeks of gestation, CL2: cervix measured between 19+0-20+6 weeks of gestation, CL3: cervix measured between 21+0-23+6 weeks of gestation.

**Table S2.** Cervical shortening between CL1-CL3 and the preterm birth risk in women with a history of spontaneous preterm birth, a shortest cervical length >25mm, and progesterone treatment or no progesterone treatment.

| **Progesterone treatment*** | | | | | | |
| --- | --- | --- | --- | --- | --- | --- |
|  | **sPTB<37 weeks of gestation**  **n=80/340** | **Odds Ratio (95%CI)** | **sPTB <34 weeks of gestation**  **n=31/340** | **Odds Ratio (95%CI)** | **sPTB <28 weeks of gestation n=6/340** | **Odds Ratio (95%CI)** |
| No shortening, n (%) n=141 | 27/141 (19.1) | 1.00 (ref.) | 9/141 (6.4) | 1.00 (ref.) | 2/141 (1.4) | 1.00 (ref.) |
| 1-10mm, n (%)  n=171 | 43/171 (25.1) | 1.42 (0.82-2.44) | 17/171 (9.9) | 1.62 (0.70-3.75) | 2/171 (1.2) | 0.82 (0.11-5.91) |
| >10 mm, n (%)  n=28 | 10/28 (35.7) | 2.35 (0.97-5.65) | 5/28 (17.9) | 3.19 (0.98-10.37) | 2/28 (7.1) | 5.35 (0.72-39.67) |
| **No progesterone treatment** | | | | | | |
|  | **sPTB<37 weeks of gestation**  **n=19/129** | **Odds Ratio (95%CI)** | **sPTB <34 weeks of gestation**  **n=11/129** | **Odds Ratio (95%CI)** | **sPTB <28 weeks of gestation n=3/129** | **Odds Ratio (95%CI)** |
| No shortening, n (%) n=52 | 8/52 (15.4) | 1.00 (ref.) | 5/52 (9.6) | 1.00 (ref.) | 2/52 (3.8) | 1.00 (ref.) |
| 1-10mm, n (%)  n=68 | 11/68 (16.4) | 1.08 (0.40-2.92) | 6/67 (9.0) | 0.93 (0.27-3.22) | 1/67 (1.5) | 0.38 (0.03-4.30) |
| >10 mm, n (%)  n=10 | 0/10 | n/a | 0/10 | n/a | 0/10 | n/a |

sPTB: spontaneous preterm birth. CL1: cervix measured between 14+0-18+6 weeks of gestation, CL3: cervix measured between 21+0-23+6 weeks of gestation. *Interventions received include progesterone (n=285 weekly intramuscular Proluton® injections, n=55 daily vaginal tablets Utrogestan®).
